# Low-Cost Microfluidic Mixers: Are They up to the Task?

**DOI:** 10.3390/pharmaceutics17050566

**Published:** 2025-04-25

**Authors:** Jade Forrester, Callum G. Davidson, May Blair, Lynn Donlon, Daragh M. McLoughlin, Chukwuebuka R. Obiora, Heather Stockdale, Ben Thomas, Martina Nutman, Sarah Brockbank, Zahra Rattray, Yvonne Perrie

**Affiliations:** 1 Strathclyde Institute of Pharmacy and Biomedical Sciences, University of Strathclyde, 161 Cathedral Street, Glasgow G4 0RE, UK; jade.forrester.2017@uni.strath.ac.uk (J.F.); callum.davidson@strath.ac.uk (C.G.D.); zahra.rattray@strath.ac.uk (Z.R.); 2 Centre for Process Innovation (CPI), Coxon Building, John Walker Rd., Sedgefield, Stockton-on-Tees TS21 3FE, UK; may.blair@uk-cpi.com (M.B.); lynn.donlon@uk-cpi.com (L.D.); daragh.mcloughlin@uk-cpi.com (D.M.M.); richard.obiora@uk-cpi.com (C.R.O.); heather.stockdale2@uk-cpi.com (H.S.); ben.thomas@uk-cpi.com (B.T.); 3 Medicines Discovery Catapult, Alderley Park, Macclesfield SK10 4ZF, UK; martina.nutman@md.catapult.org.uk (M.N.); sarah.brockbank@md.catapult.org.uk (S.B.)

**Keywords:** lipid nanoparticle, mRNA, microfluidic mixing, manufacture, characterization

## Abstract

**Background/Objectives**: Microfluidic mixing has become the gold standard procedure for manufacturing nucleic acid lipid-based delivery systems, offering precise control over critical process parameters. The choice and design of microfluidic mixers are often seen as a key driving force affecting the critical quality attributes of the resulting lipid nanoparticles (LNPs). **Methods**: This study aimed to evaluate LNPs manufactured using two low-cost microfluidic mixers alongside manual mixing (pipette mixing (PM)), followed by characterization studies using orthogonal analytics as well as expression studies to establish whether low-cost microfluidic manufacturing methods are suitable for bench-scale and high-throughput research. **Results**: The results show that all manufacturing methods can produce LNPs with sizes ranging between 95 and 215 nm with high encapsulation (70–100%), and enhanced analytics showed variations between the LNPs produced using the different mixers. Despite these differences, pipette mixing production of LNPs demonstrated its application as a high-throughput screening tool for LNPs, effectively distinguishing between different formulations and predicting consistent expression patterns both in vitro and in vivo. **Conclusions**: Overall, these results validate the use of low-cost microfluidic mixers without compromising the efficiency and integrity of the resulting LNPs. This study supports the increased accessibility of small-scale LNP manufacturing and high-throughput screening.

## 1. Introduction

Traditionally, lipid nanoparticles and other nanoparticle delivery systems were commonly manufactured using complex techniques, often resulting in large particles requiring additional steps to reduce size, making these techniques hard to scale up and costly to maintain [1]. As nanoparticle technology has advanced, so too have manufacturing methods. In an effort to create user-friendly, cost-effective and scalable nanoparticle manufacturing methods, microfluidic mixing was developed. Microfluidic mixing can briefly be described as the mixing of two or more fluids within a microscale environment [2], with advantages including higher encapsulation efficiencies, the possibility to incorporate both lipophilic and hydrophilic drugs, and an overall decreased cost and time constraint [3,4]. Microfluidics results in nanoparticle formation in a single-step process involving the mixing of an aqueous phase (commonly including the moiety to be entrapped dissolved in buffer, e.g., mRNA in the case of LNPs) with a lipid phase (dissolved in solvent, e.g., ethanol). Indeed, microfluidics is now routinely involved in LNP manufacture, and Pratsinis et al. demonstrated using a Design of Experiments (DoE) approach that when compared to solvent injection methods, microfluidics resulted in small LNPs with a more homogenous population [5]. Manufacturing using microfluidic devices can involve either active mixing (requiring external forces, e.g., vibration or electrical forces) or passive mixing (e.g., cross-flow or flow-focusing), which will additionally have variations in internal structures and diameters of mixers. This, in turn, can influence the critical quality attributes (CQAs) of the resulting LNP formulations [6,7,8]. The degree of mixing can also be influenced by the channel length and contact surface area of the liquid/liquid interface [8].

**Figure 1:**
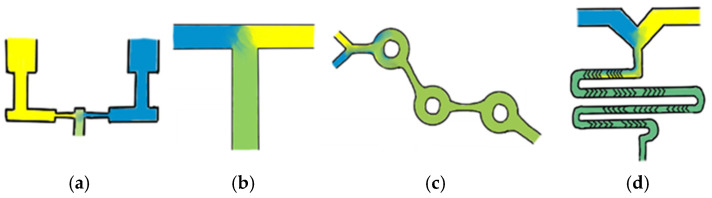
A simplified schematic of the common microfluidic mixers. Representations of the flow in common microfluidic mixers: (**a**) confined impingement jet mixer, (**b**) T-junction mixer, (**c**) toroidal mixer, and (**d**) staggered herringbone mixer. Image prepared using Sketchbook v6.1.

One of the earliest and easiest to use microfluidic mixer designs is a standard T-junction mixer, which typically involves the aqueous and lipid phases being injected directly into each other at high speeds, resulting in the formation of LNPs. This method often enables precise control over size; however, it requires increased waste volumes to allow for fluid mixing equilibration, making it less suitable for small-scale research. Additionally, the high forces generated in these mixers can disrupt droplet formation at the interface, potentially affecting LNP uniformity and stability [8,9]. Despite this, T-junction mixers have demonstrated significant clinical success, with Pfizer BioNTech manufacturing their COVID-19 vaccine using a proprietary T-mixer design [10]. A second common design, known as impinging jet mixers, features a set-up in which fluids are constricted and directed toward each other at high speeds, resulting in nanoprecipitation. These mixers can be particularly useful when dealing with higher viscosity liquids. However, similar to T-mixers, these mixers are subject to high shear forces [7]. Despite these challenges, impinging jet mixers help overcome the complexities of fluidic control through fewer fluid channels and a simpler design [6]. As LNP systems have gained popularity, the demand for precise control over LNP CQAs, coupled with enhanced scalability, has increased. Mixers that excel in these parameters often harness chaotic flow, differing from traditional methods by incorporating groves or advections that disrupt laminar flow [3,7]. One example of a chaotic flow mixer is the staggered herringbone mixer (SHM), which contains two inlet channels flowing into a mixer with ‘v’ shaped advections, causing transverse flow and enhancing the mixing of the two fluids. Another example is the toroidal mixer (TM), which causes fluids to wrap around each other due to circular flow path structures [7,8,11]. Despite their advantages, industry-leading chaotic mixers are often expensive, rely heavily on single-use plastics, and are not the only viable option for producing small, homogenous LNP populations. Simplified schematic representations of these microfluidic mixers are shown in Figure 1; however, these mixer designs encompass only a small portion of the diverse range of available mixing methods.

Overall, different mixer designs can produce LNPs with varied CQAs, making it essential to understand how each mixer type influences nanoparticle characteristics to successfully select the most appropriate system. Beyond mixer design, the internal size of the mixer also plays a crucial role in LNP formation. For example, Maeki et al. conducted a study in which they designed two chaotic advection mixers with different channel depths and demonstrated that, for a liposomal formulation, a larger channel depth resulted in higher mixing efficiency [12]. Additionally, passive and active mixers can be combined to refine process parameters further. For instance, Peng et al. coupled ultrasonic microreactors with either co-flow or confined impingement jet mixers to manufacture liposomal formulations, illustrating the potential of hybrid approaches [13]. Furthermore, key process parameters, such as the speed at which the fluids are delivered (total flow rate; TFR) and the ratio at which the fluids are delivered (flow rate ratio; FRR), are vital in controlling the CQAs of LNPs. For example, McMillan et al. and Roces et al. both demonstrated that varying FRRs offered a precise method for controlling LNP size [14,15].

Here, we briefly highlighted a small portion of the extensive diversity of microfluidic mixer design. This study aims to determine whether low-cost, reusable microfluidic mixers can produce LNPs within a standard target CQA range with high payload entrapment and preclinical efficacy to allow for the low-cost and rapid screening of LNP formulations. To do this, two microfluidic mixing devices, a T-junction mixer (TM) and a confined impingement jet mixer (IJM), both equipped with automated syringe drivers, alongside a manual mixing process (pipette mixing (PM)), were used to manufacture LNPs. These LNPs were then subjected to orthogonal analysis techniques and preclinical in vitro and in vivo testing. A high throughput pipette mixing robot was also employed to assess PM as an LNP screening and optimization platform. Overall, this study aimed to establish if low-cost microfluidic mixers can reliably and reproducibly produce LNPs suitable for screening and testing of LNPs.

## 2. Materials and Methods

### 2.1. Materials

The ionizable lipids Heptadecan-9-yl 8-((2-hydroxyethyl)[6-oxo-6-(undecyloxy)hexyl]amino) octanoate (SM-102) and (6Z, 9Z, 28Z, 31Z)-Heptatriaconta-6,9,28,31-tetraen-19-yl 4-(dimethylamino)butanoate (D-Lin-MC3-DMA) were purchased from Broadpharm (San Diego, CA, USA). 1,2-dimyristoylrac-glycero-3-methoxypolyethylene glycol-2000 (DMG-PEG2000), 1,2-distearoyl-sn-glycero-3-phosphocholine, 1,2-dioleoyl-3-trimethylammonium-propane (DOTAP), and 1,2-dioleoyl-3-dimethylammonium-propane (DODAP) were acquired from Avanti Polar Lipids (Alabaster, AL, USA). Cholesterol, polyadenylic acid (poly A), heat-inactivated fetal bovine serum (FBS) and HPLC-grade methanol were purchased from Merck Life Science UK Ltd. (Gillingham, UK). Ethanol was purchased from Suppelco (Bellefonte, PA, USA). Sodium citrate buffer, Tris-HCL buffer, Triethylamine acetate buffer, propidium iodide, Trypan Blue, and the Quanti-iT RiboGreen RNA Assay kit were purchased from Thermo Fisher Scientific (Invitrogen, Thermo Fisher Scientific, Waltham, MA, USA). EZ Cap^TM^ Firefly Luciferase mRNA (5-moUTP) and EZ Cap^TM^ Cy5 Firefly Luciferase mRNA (5-moUTP) were purchased from APExBIO (Houston, TX, USA). CPI (Centre for Process Innovation, Sedgefield, UK) supplied Green Lantern mRNA. A Rabbit Reticulocyte Lysate kit, ONE-Glo™ Luciferase Assay System, and VivoGlo™ Luciferin were acquired from Promega Ltd. (Chilworth, UK). Dulbecco’s Minimal Essential Media (DMEM), Minimal Essential Media (MEM), sodium pyruvate, penicillin/streptomycin, and TrypLE were purchased from Gibco Technologies. Ultrapure water was supplied through an in-house system. HEK-293 cells were purchased from American Tissue Culture Collection (ATCC) (CRL-1573).

### 2.2. Microfluidic Mixer Set-Up

The T-junction mixer (TM) consisted of a 3-port manifold T-junction (Darwin Microfluidics); aqueous and lipid phase inlets flow directly into each other while the LNP outlet is positioned perpendicularly. The impingement jet mixer (IJM) consisted of a 5-port confined impinging jet mixer (CIJM) (Holland Applied Technologies, Burr Ridge, IL, USA); 3 of the 5 ports were used with the aqueous and lipid phase inlets flowing down, then directly into each other, with the LNP outlet positioned perpendicularly for sample collection. Both microfluidic mixers were combined with World Precision Instrument AL-1010 syringe pumps, which were automated using the SyringePumpPro software v1.7.0.0 to allow for precise control over mixing speeds and ratios. Pipette mixing (PM) was accomplished using Gilson P1000 and P200 pipettes (Dunstable, UK).

### 2.3. Manufacture of Lipid Nanoparticles (LNPs)

The aqueous phase consisted of poly A, Fluc mRNA, Green Lantern mRNA, or Cy5-Fluc mRNA diluted in 50 mM pH 5 citrate buffer to account for an N/P ratio of 6. The lipid phase consisted of DSPC, cholesterol, ionizable/cationic lipid (SM-102, D-Lin-MC3-DMA, DODAP, or DOTAP) and DMG-PEG2000 at a molar ratio of 10:38.5:50:1.5% dissolved in absolute ethanol (≥99.5%). LNPs were manufactured on the TM, IJM, or PM to achieve LNPs with a final payload concentration of 0.04 mg/mL and a final lipid concentration of 0.9 mg/mL. For TM and IJM, total flow rates (TFRs) were maintained at 15 mL/min, while the flow rate ratio (FRR) was kept constant at 3:1 (aqueous/lipid) for all mixers.

### 2.4. Purification of LNPs

Subsequent LNPs were purified using either dialysis or spin column chromatography. Dialysis purification consisted of LNPs being dialyzed against 10 mM pH 7.5 Tris buffer for 4 h using dialysis tubing with a molecular weight cut-off of 14 kDa. Spin column purification was carried out with LNPs being diluted 1:40 with 10 mM pH 7.5 Tris buffer, and centrifugal filtration was carried out using Amicon Ultra-15 columns at 2000× *g* 4 °C for approximately 10 min or until the desired volume was achieved.

### 2.5. LNP Characterization

The critical quality attributes (CQAs) of LNPs were monitored using Dynamic Light Scattering (DLS)/Electrophoretic Light Scattering (ELS). The z-average diameter (nm), polydispersity index, and zeta potential (mV) were measured using the Zetasizer Ultra (Malvern Panalytical, Ltd., Worcestershire, UK). The LNPs were diluted to a 0.1 mg/mL lipid concentration for analysis. Pre-purification measurements were diluted in citrate buffer (viscosity 1.28 cP and refractive index 1.47), post-purification measurements were diluted in Tris buffer (viscosity 1.0037 cP and refractive index 1.337), and zeta potential measurements were diluted in water (viscosity 0.8872 cP and refractive index 1.33).

Payload encapsulations were quantified using the Quanti-iT^TM^ RiboGreen^TM^ Assay Kit (Invitrogen, Thermo Fisher Scientific, Waltham, MA, USA. High (0–1000 ng) and low (0–50 ng) curves were prepared using the encapsulated payload to quantify the encapsulated material from Triton-treated and non-Triton-treated wells. Briefly, samples were diluted to a concentration of 3 μg/mL payload and mixed with either 50 μL of 2% Triton-TE or 1× TE buffer before being incubated for 15 min at 37 °C. Following on, a 200× and 500× RiboGreen solution was added to the Triton-treated and non-Triton-treated wells, respectively, to account for a 1:1 dilution. The fluorescence was then measured using the Glomax (Promega, Chilworth, UK) at excitation 475 nm and emission 500–550 nm.

### 2.6. Nanoparticle Tracking Analysis

Nanoparticle Tracking Analysis (NTA) was conducted using the NS300 equipped with a 488 nm laser block (Malvern Panalytical, Worcestershire, UK). Samples were diluted 1:5000 (PM) or 1:2500 (TM/IJM) in Tris buffer. Each sample was measured five times for 60 s at ambient temperature, with flow settings at a constant setting of 200, with a camera level of 9 and a screen gain of 6. Processing analysis was conducted using a detection threshold of 4, with the software version NTA 3.4 Build 3.4 003. The span parameter was calculated using ((D90-D10)/D50).

### 2.7. In Vitro Protein Expression of LNPs

HEK-293 cells were seeded at 1.5 × 10^4^ cells/well in either a 96-well white (Fluc) or black (Green Lantern) clear bottom plate in Minimal Essential Media (MEM) (supplemented with 10% FBS, 5% sodium pyruvate, and 5% penicillin streptomycin) and incubated at 37 °C (5% CO_2_) for 48 h. Following incubation, cells were treated with LNPs serially diluted to achieve mRNA concentrations of 2–0.25 μg/mL and left to incubate for a further 48 h at 37 °C (5% CO_2_). For Fluc-LNPs, to measure results, 100 μL of One-Glo luciferase was added to each well, and luminescence was measured using the Glomax (Promega, Chilworth, UK). For Green Lantern-LNPs, the fluorescence was measured using the Polarstar Omega Plate Reader (BMG LABTECH Ltd., Aylesbury, UK).

### 2.8. Measuring LNP Uptake Using Flow Cytometry

For LNP uptake, cells were plated in a 12-well plate at a concentration of 5 × 10^5^ cells/well and incubated for 48 h at 37 °C (5% CO_2_). Then, 24 h pre-analysis, the dead cell control was treated with 0.1% Triton-MEM and 4 h pre-analysis, LNPs were added to the appropriate well at a mRNA concentration of 2 μg/mL. Sample preparation was carried out at room temperature, spent media was removed from wells and cells were washed with DBPS; TrypLE was added to all wells and cells were allowed to dissociate at 37 °C (5% CO_2_) for 2 min. After this, MEM was added to all wells, and samples were spun at 300× *g* for 5 min until a cell pellet was observed. The supernatant was removed, and the cell pellet was resuspended in DPBS, and where appropriate, 2 drops of propidium iodide were added. Samples remained at room temperature for 15 min before being transferred onto ice until analysis was complete. Flow cytometry measurements were done using the Attune NxT Flow Cytometer (Thermo Fisher) using the BL2 laser to monitor PI and the RL1 laser to monitor Cy5 fluorescence.

Flow cytometry analysis was gated based off of three control groups, unstained live cells (Figure S1), stain live cells (Figure S2) and stained dead cells (Figure S3), once gating was appropriate for all three controls, analysis of samples was undertaken.

### 2.9. In Vivo Protein Expression Studies

For comparing mixers, 14-week-old female BALB/c mice were intramuscularly injected with 50 μL/hind leg of LNPs, equating to 2 μg total Fluc mRNA. Then, 0.5 h post-injection, mice were subcutaneously injected with 100 μL of Vivo-Glo Luciferin, and after 10 min, bioluminescence signals were captured using an IVIS Spectrum imaging system (Revvity, Inc., Waltham, MA, USA). Bioluminescence signals were further captured at 0.5-, 6- and 24-h post-injection. For comparing formulations, 8-week-old male BALB/c mice were intramuscularly injected with 50 µL/hind leg of LNPs, equating to 2 µg total Fluc mRNA. Bioluminescence signals were captured as mentioned above, at 6- and 24-h post-injection. Bioluminescence signals were quantified using the Living Image 4.0 Software.

### 2.10. Cell-Free Protein Expression (CFPE)

A Rabbit Reticulocyte Lysate (RRL) system was used to analyze CFPE. Briefly, Fluc mRNA was extracted from LNPs by mixing 200 μL of sample with 750 μL ethanol and 25 μL sodium acetate at 3 M pH 5, before being centrifuged at 14,000 rpm for 20 min. Ethanol precipitation was carried out twice, and the resulting mRNA pellet was resuspended in 35 μL of RNAse-free water. Extracted mRNA was then used to complete reaction mixtures consisting of 35 μL rabbit reticulocyte lysate, 0.5 μL of both amino acid mixtures minus leucine or methionine, 1 μL RNasin^®^ Ribonuclease Inhibitor, 2 μL of extracted mRNA, control mRNA (positive) or no mRNA (negative), and made up to a final volume of 50 μL with ultrapure water. The reaction mixtures were incubated at 30 °C for 1.5 h, and the reaction was terminated by placing them on ice. To measure protein expression, reaction mixtures were mixed with an equal volume of ONEGlo luciferase and luminescence was measured on the Glomax (Promega).

### 2.11. High Performance Liquid Chromatography–Evaporated Light Scattering Detector (HPLC-ELSD)

Lipid recovery was measured using the Shimadzu HPLC-ELSD III system. LNPs were injected at a volume of 10 μL at a flow rate of 0.5 mL/min, and lipids were separated using a Waters Column XBridge^®^ C18 Column (internal diameter 3 mm, column length 150 mm, pore size 130 Å, and 3.5 μm particle size) with a detection temperature of 50 °C and a column oven temperature of 45 °C. Mobile phase A consisted of 0.01 M Triethylamine acetate and water, while mobile phase B consisted of 0.01 M Triethylamine acetate and ethanol. The run time was 15 min with a binary gradient, with mobile phase B increasing from 70 to 100% from 0 to 7 min with a drop back to 70/30% B/A gradient at 12.1 min. Samples were diluted 1:5 (*v*/*v*) with mobile phase B before analysis. Concentration curves were developed for each lipid, and all analysis was done using the Shimadzu post-run software v5.124 SP1.

### 2.12. Frit-Inlet Asymmetric Flow Field-Flow Fractionation (FI-AF4) Multiplexed with Multiangle Light Scattering, Dynamic Light Scattering, and UV Detection

The asymmetric flow field-flow fractionation module (AF2000, Postnova Analytics, Malvern, UK) was hyphenated with an inline multidetector (MD), multiangle light scattering (MALS-PN3621, Postnova Analytics), UV detector (PN3242, 260 nm, 0.5 sensitivity—Postnova Analytics), and DLS Zetasizer Nano ZS System (Malvern Panalytical, Worcestershire, UK). A frit-inlet channel (300 mm × 60 mm × 40 mm) used a 350 μm spacer, 10 kDa MWCO amphiphilic regenerated cellulose membrane and 10 mM pH 7.5 Tris as the eluent liquid for separation at 25 °C. The FI-AF4-MD methodology was used from Davidson et al. [16] with a cross-flow (XF) initial hold of 15 min at 0.75 mL/min, followed by a 60 min exponential XF decay from 0.75 mL/min to 0 mL/min and a hold of 0 mL/min for 5 min; throughout separation, a detector flow (DF) of 0.3 mL/min was used.

PM, TM, and IJM LNP samples were diluted to 0.5 mg/mL lipid concentration in eluent liquid prior to analysis. A 20 μL sample was injected using a 100 μL injection loop. The sample recovery, radius of gyration (Rg) from the MALS detector, hydrodynamic radius (Rh) from the inline DLS, and shape factor were calculated as per Abdulrahman et al. [17]. The collected MALS data were analyzed using the AF2000 software v2.2.01 (Postnova Analytics, Germany), which uses a spherical fit model across 52°–124° angles.

### 2.13. High-Throughput Formulation of LNPs Using Automated Platform

The aqueous phase consisted of Green Lantern mRNA diluted in 5 mM pH 4.5 citrate buffer. The mRNA concentration was kept at 0.05 mg/mL in all formulations. The lipid phase again consisted of DSPC, cholesterol, and DMG-PEG2000 and SM-102, D-Lin-MC3-DMA, DODAP, or DOTAP at a molar ratio of 10:38.5:50:1.5% dissolved in ethanol. The lipids and mRNA solutions were mixed in a robotic liquid handling system (JANUS G3 automated workstation). A robotic liquid handling system prepared the lipid mixture and mRNA dilutions for the high-throughput process. The mixing volume ratio of the aqueous/lipids in ethanol was maintained at 3:1, and the pipetting speed was set at 600 µL/s. LNP formulations were diluted 10-fold in Tris sucrose buffer to achieve a final concentration of 3.75 µg/mL mRNA.

### 2.14. Physicochemical Characterization of LNPs by Automation

Critical quality attributes (CQAs) of LNPs were monitored using Dynamic Light Scattering (DLS). The z-average diameter (nm) and polydispersity index of LNPs were measured in a 96-well plate using a DynaPro plate reader II (Wyatt Technology UK Ltd., Wilmslow, UK). LNPs were diluted 10-fold in Tris buffer using a CyBio FeliX liquid handler (Analytik Jena UK Ltd., London, UK) for size measurement. Solvent parameters for sucrose 2% were applied for analysis, including a viscosity of 1.055 cP and refractive index of 1.336 (25 °C).

The payload encapsulation efficiency (EE%) was quantified using the Quanti-iT^TM^ RiboGreen^TM^ RNA Assay Kit (Invitrogen^TM^) following the manufacturer’s instructions and adapted to be performed on a CyBio FeliX liquid handler (Analytik Jena), integrated with a PreciseFlex 750 robotic arm, STX44 incubator (LiCONiC, Montabaur, Germany), labware hotel, barcode reader, and Synergy H1 microplate reader (BioTek, Winooski, VT, USA). A high (0–1000 ng) standard curve was prepared using the encapsulated payload to quantify RNA from Triton-treated and non-Triton-treated wells. Briefly, samples were diluted to a concentration of 1 µg/mL mRNA and mixed with 50 µL of either 2% Triton-TE or 1× TE buffer before incubation at 37 °C for 15 min. Following incubation, a 200× RiboGreen solution was added to all wells for a final 1:1 dilution. Fluorescence was then measured at an excitation of 485 nm and an emission of 535 nm.

### 2.15. High-Throughput In Vitro Assay

A high-throughput cell-based in vitro assay for interrogation of LNP efficacy was developed by the IDDC consortium to support formulation screening. LNPs encapsulating mGreenLantern mRNA were dosed using an Echo liquid handler at 1 ng, 10 ng, or 30 ng mRNA per well in a 384-well plate (Greiner µCLEAR^®^, Nürtingen, Germany). HeLa cells, which were previously modified by lentiviral transduction to express nuclear-localized mKate2, were seeded on top of LNPs at 2 × 10^3^ cells per well in FluoroBrite DMEM (Gibco, Waltham, MA, USA) containing 10% fetal bovine serum (Sigma, St. Louis, MO, USA), GlutaMAX™ Supplement (Gibco) and 1% penicillin–streptomycin (Sigma), and incubated at 37 °C (5% CO_2_) for 42 h. Following incubation, cells were fixed with 4% paraformaldehyde and imaged using an Opera Phenix high-content screening system equipped with a 10 × 0.3 NA air objective and 488 nm and 561 nm laser lines. Images were analyzed in Harmony. Automated segmentation pipelines were constructed to identify nuclei and cytosolic mGreenLantern expression with single-cell resolution. The cell number was defined as the number of detected nuclei per well, and transfection efficiency as the proportion of these cells positive for mGreenLantern expression. Data were normalized to internal controls before plotting in Microsoft Excel (Version 16.84, Microsoft Corporation, Redmond, WA, USA).

### 2.16. Cryogenic Transmission Electron Microscopy

Fluc-LNP samples were prepared as normal and then concentrated to 0.08 mg/mL mRNA. Glow discharge was carried out on Lacey carbon film, 300 mesh copper grids (Agar Scientific, Rotherham, UK) for 30 s at 30 mA using the Emitech K100X (Quorum Technologies Ltd., Laughton, UK). A total of 3 μL of the LNP samples were blotted on either Whatman 597 paper using the Thermo Fisher Vitroblot Mk IV (25 °C, 95% humidity, blot time 3 s, and blot force 13 or 12) or Whatmann 1 paper using the Leica EM GP2 plunge-freezer before being plunge frozen in liquid ethane. Samples were cooled in liquid nitrogen and held in a Gatan Elsa cryo-specimen holder until being transferred to the JEOL F2-Cryo (200 kV) with Direct Electron DE-20 camera for image capture. The acquisition software was SerialEM version 4.1, with images being processed on Relion 5.0.0 and Image J.JS v0.5.8.

### 2.17. Ethics Statement

Animal experiments and experimental procedures were carried out in accordance with UK Home Office regulations and the University of Strathclyde Animal Welfare and Ethical Review Board regulations under project license number PPL PP1650440. BALB/c mice were bred and maintained in the Biological Procedures Unit at the University of Strathclyde, Glasgow, and experiment design and reporting adhered to the ARRIVE guidelines.

### 2.18. Statistical Analysis

All results represent the mean ± standard deviation of three independent replicates. Statistical significance was determined using an ANOVA with Tukey’s post hoc test using Minitab software (v19.2). For in vivo studies, 2–3 mice were used for each condition to give 4–6 regions of interest for the analysis of the injection site and 2–3 regions of interest for the analysis of livers, with results representing the mean ± standard error of the mean (SEM). No data points were excluded.

## 3. Results

### 3.1. Physicochemical Properties of LNPs Are Dependent on the Payload Encapsulated

**Figure 2:**
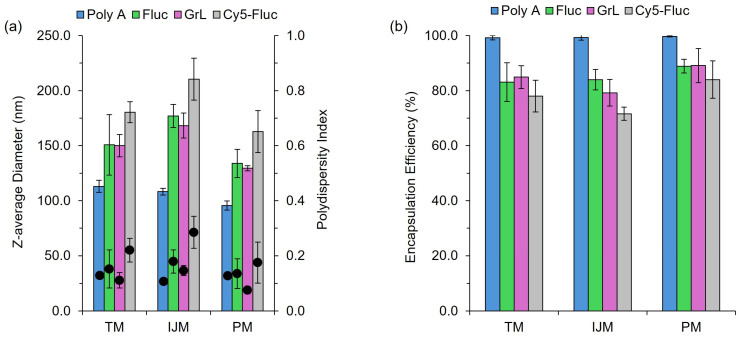
The effect of payload on LNP CQAs. LNPs composed of DSPC:chol:SM-102:DMG-PEG2000 (10:38.5:50:1.5%) encapsulating varying nucleic acid payloads. (**a**) demonstrates the z-average diameter (nm) (bars) and polydispersity index (circles). (**b**) shows the encapsulation efficiency (%). Results represent the mean ± standard deviation (SD) (*n* = 3–4). TM: T-junction mixer, IJM: confined impingement jet mixer; PM: pipette mixing, Fluc: Firefly Luciferase mRNA, GrL: Green Lantern mRNA.

A vital component of LNP-based formulations is the nucleic acid payload, with particular emphasis on how variations in the payload can affect the CQAs of the resulting LNPs. Throughout this study, four nucleic acid payloads were used: poly A, Firefly Luciferase mRNA (Fluc), Green Lantern mRNA (GrL) and Cy5-labelled Firefly Luciferase mRNA (Cy5-Fluc). The results in Figure 2 and Table 1 show that switching between these payloads led to variations in CQAs, highlighting the impact of payload selection on LNP characteristics.

Figure 2a highlights that, regardless of the manufacturing method used, particle size and polydispersity (PDI) differences can be seen depending on the nucleic acid payload. For all mixers, poly A-LNPs were significantly (*p* < 0.05) smaller in size compared to the other formulations, while Cy5-Fluc-LNPs resulted in LNPs significantly larger (*p* < 0.05) compared to the other LNPs. In contrast, varying between mRNA constructs (Fluc-LNPs vs. GrL-LNPs) had no significant effect. Cy5-Fluc-LNPs also exhibited a significant increase in PDI (*p* < 0.05) when manufactured using the TM or IJM methods. For Fluc-, GrL- and Cy5-Fluc-LNPs, manufacturing on the IJM also resulted in significantly (*p* < 0.05) larger particle sizes. For LNPs manufactured on either the TM or IJM, a significant (*p* < 0.05) decrease in encapsulation efficiency was seen for LNPs encapsulating mRNA compared to LNPs encapsulating poly A (Figure 2b). The results in Table 1 indicate that all mixing methods produced LNPs with zeta potential values near neutral across all nucleic acid payloads, with no significant differences observed between mixers.

### 3.2. Low-Cost Microfluidic Mixers Can Produce LNPs with Sufficient In Vitro Protein Expression

**Figure 3:**
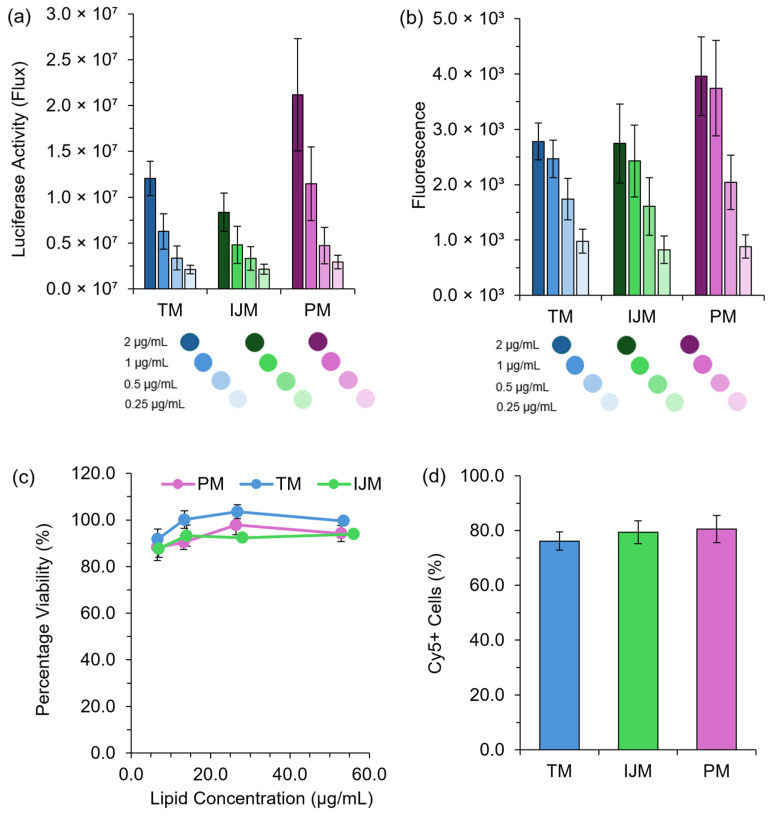
In vitro performance of LNPs. HEK-293 cells were treated with either Fluc-LNPs or GrL-LNPs at mRNA concentrations of 2 μg/mL to 0.25 μg/mL, and luminescence signals (**a**) or fluorescence signals (**b**) were monitored 48 h after transfection as a direct measure of protein expression. (**c**) demonstrates the percentage viability of HEK-293 cells 48 h after transfections with GrL-LNPs, while (**d**) demonstrates the percentage of Cy5+ cells 4 h after transfection with Cy5-Fluc-LNPs measured using flow cytometry. All results are representative of the mean ± standard error of the mean (SEM) (*n* = 3).

LNP in vitro efficiency was determined using HEK-293 cell models with either a Fluc or GrL mRNA construct. Figure 3 highlights that all LNPs had similar expression, viability, and uptake regardless of the microfluidic mixer used.

Regardless of the mRNA construct used, LNPs manufactured using TM, IJM, and PM all promoted in vitro protein expression (Figure 3a,b), with no significant difference observed between the formulations. No toxicity issues were noted across all treatment concentrations and mixing methods tested (Figure 3c). To assess cellular uptake, LNPs encapsulating Cy5-Fluc were manufactured and used as a direct uptake indicator. Figure 3d shows that four hours post-transfection, all LNP formulations exhibited high uptake in HEK-293 cells, with ~80% Cy5+ cells observed across all mixers, confirming efficient cellular internalization.

### 3.3. Determining Lipid and mRNA Integrity of LNPs

**Figure 4:**
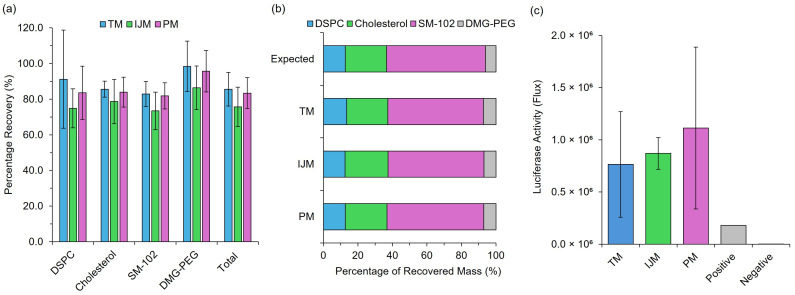
Post-manufacture integrity monitoring of LNPs. (**a**) represents the individual and total lipid recovery measured using Shimadzu HPLC-ELSD III of GrL-LNPs, while (**b**) represents the respective recovery in terms of the mass ratio of lipids. (**c**) represents the luciferase activity (flux) as a direct measure of the protein expression produced from mRNA extracted from Fluc-LNPs and tested in a Rabbit Reticulocyte Lysate cell-free protein expression system. Results represent the mean ± standard deviation (SD) (*n* = 3).

Post-manufacture monitoring of LNPs is essential for ensuring the stability and efficacy of LNP-based formulations. While CQAs such as particle size play a crucial role in formulation performance, assessing the integrity of the lipid and payload components is equally important. To achieve this, in addition to testing in vitro and in vivo efficacy, a cell-free protein expression system was employed to evaluate mRNA expression levels following extraction from LNPs, while HPLC-ELSD analysis was used to assess lipid integrity and ensure high lipid recovery (Figure 4).

Regardless of the manufacturing method used for LNPs, all formulations had total recoveries greater than 75%, with individual lipid recoveries ranging between 73 and 100% (Figure 4a), and all formulations resulted in mass ratios similar to the expected mass ratio—varying with <2% error across lipids (Figure 4b). This ensures that, although some product loss is inevitable, it occurs at the same rate across all lipid components. Unequal loss, particularly of critical components such as the ionizable lipid, could significantly compromise LNP functionality. Finally, extracted mRNA from the formulations was then used to treat CFPE systems at a concentration of 2 μg/mL, and this showed similar results to in vitro testing with no significant differences in expression levels (Figure 4c). These results confirm that low-cost microfluidic mixers do not compromise the integrity or functionality of LNPs.

### 3.4. Pipette-Mixed LNPs Exhibit Comparable In Vivo Protein Expression to Low-Cost Microfluidic Mixers

**Figure 5:**
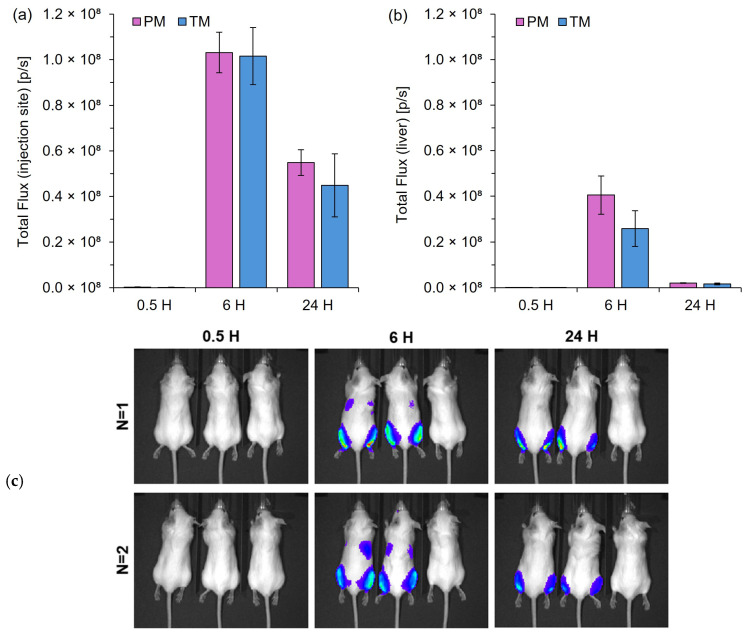
In vivo protein expression following IM injection of mRNA-LNPs prepared by pipette mixing (PM) or T-junction mixing (TM). Quantification of total flux at the injection site (**a**) and liver region (**b**) at 0.5, 6, and 24 h post-injection. (**c**) Representative images of the BALB/c mice (mouse order from left to right: PM, TM, and control mouse). The color scale represents the luminescence intensity in radiance (*p*/s/cm^2^/sr), ranging from 0.5 × 10^7^ (blue) to 2.0 × 10^7^ (red); the minimum and maximum values recorded were 7.0 × 10^5^ and 1.8 × 10^7^ p/s/cm^2^/sr, respectively. Bioluminescence signals were quantified using the Living Image 4.0 software, with regions of interest defined at the injection point (**a**) and liver (**b**). Results are expressed as the mean ± standard error of the mean (SEM) (*n* = 4 for leg; *n* = 2 for liver).

For in vivo protein expression, BALB/c mice were treated with LNPs encapsulating Fluc mRNA and imaged on the IVIS Spectrum (Figure 5). Due to the similar CQAs between TM and PM samples, both LNPs were included in the in vivo studies to determine whether manual mixing had any significant effect on the protein expression of the LNPs.

Figure 5 highlights that LNPs made via a manual pipette mixing process (PM) with no control over speed could produce protein expression levels similar to that of an automated controlled T-junction mixer (TM) with no statistically significant differences. Protein expression levels were highest 6 h post-injection, with sustained expression evident 24 h post-injection (Figure 5a). Moderate levels of expression can also be seen in the liver 6 h post-injection, but were minimal at the 24 h time point (b). The results in Figure 5 highlight PM as an easy, quick, and cheap alternative to microfluidic mixing in bench-scale research.

### 3.5. Orthogonal Analysis of LNPs

**Figure 6:**
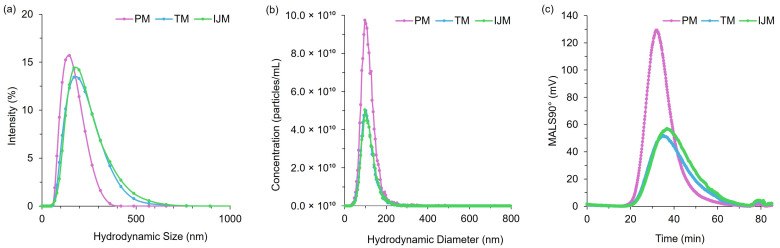
Distribution profiles for PM, TM, and IJM Fluc-LNPs: (**a**) hydrodynamic size by DLS (intensity distribution), (**b**) hydrodynamic size by NTA (particle concentration, particles/mL) and (**c**) MALS 90° signal by AF4-MALS. Data represent averages from two independent runs per method, comprising ten measurements (NTA), six measurements (DLS), and six (PM/TM) or five (IJM) injections (MALS).

**Figure 7:**
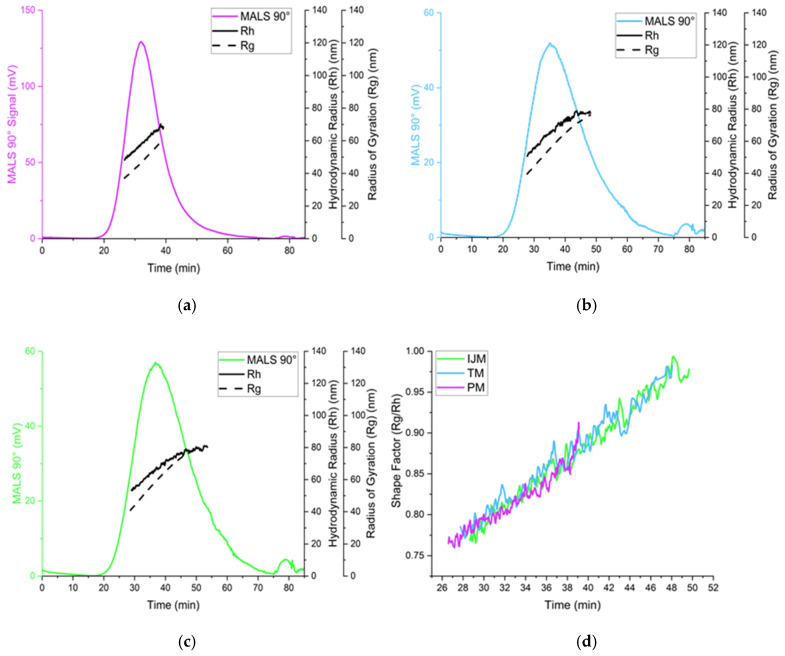
Analysis of Fluc-LNPs manufactured on either the PM (**a**), TM (**b**), or IJM (**c**), represented by the scattering intensity at the MALS 90° scattering angle (colored lines), hydrodynamic radius (Rh) (black line), and radius of gyration (Rg) (dotted black line). (**d**) represents the shape factor over time for all three manufacture methods. Results are representative of the mean ± standard deviation (SD) of two independent repeats consisting of six (PM/TM) or five (IJM) injections.

While PM, TM, and IJM showed no statistically significant variations in vitro, and PM and TM demonstrated comparable in vivo efficacy, further detailed orthogonal analyses were conducted to investigate physicochemical differences between LNPs produced using different mixers. To achieve this, three complementary techniques were employed. Dynamic Light Scattering (DLS) provided a rapid average measurement of a population’s physicochemical properties, including size and polydispersity, as shown in Table 2. Nanoparticle Tracking Analysis (NTA) provides a more detailed assessment of individual particle sizes and concentrations, reporting results as number-based rather than intensity-based values. This enables measurement of the size and polydispersity of the nanoparticle population without larger particles dominating the intensity signal and skewing particle size average; these results are presented in Table 3 and the distribution plots are shown in Figure 6. Asymmetric flow field-flow fractionation (AF4) with inline detectors offers enhanced resolution by separating particles based on the Stokes–Einstein equation. This technique allows for the analysis of additional characteristics, such as the radius of gyration (Rg). The Rg reflects the distribution of the mass within a particle and, when coupled with the hydrodynamic radius (Rh), can give insights into the shape factor of the LNPs, indicating whether they have a spherical shape. These results are detailed in Table 4 and Figure 7. By employing these orthogonal techniques, key physicochemical properties, including size, polydispersity, distribution profiles, Rg, particle concentration, and shape factor, were examined to better understand the influence of mixer selection on LNP characteristics.

The use of the orthogonal techniques highlights the range of size and heterogeneity information that can be collated for LNPs, and while all the methods provide different readouts, the pattern of PM producing smaller, more monodisperse particles compared to TM or IJM remained the same (Table 2, Table 3 and Table 4). Overall, it was shown that the AF4 method was successful across all mixers, with formulations reaching a recovery of ~80% for TM and ~90% for both PM and IJM, which aligned with the ISO standard [18] (Table 4). PM demonstrated the largest MALS 90° signal with minimal sub-populations, indicating enhanced diffusion and separation within the channel. This could be attributed to the increased particle concentration, as confirmed by the NTA. Results from the NTA showed less variation in particle size across the mixers when compared to DLS due to NTA generating a number-based measurement rather than an intensity-weight distribution. Regardless of this, PM LNPs consistently showed narrower particle sizes and more monodisperse populations across DLS, NTA, and AF4 (Figure 6). On the other hand, TM and IJM produced wider MALS 90° peaks with an increased number of shouldering peaks, indicating a higher presence of sub-populations (Figure 7a,b). TM and IJM also exhibited wash-off of aggregates when the crossflow reached 0 mL/min, eluting as a small peak after 75 min, which is during the membrane wash, indicating wash-off of unseparated larger aggregates (Figure 7b,c). Measurement of the ratio between Rg and Rh values also allowed for the shape factor of the LNPs to be analyzed, where it was seen that all LNPs exhibited similar spherical shape factors of ~0.775. Due to the shorter and wider area of the TM and IJM peaks, these LNPs have an increased FWHM, leading to increased shape factors as time increases, indicating possible oval or conjoined string shapes for LNPs (Figure 7d). Finally, the Rg-span from AF4 and span calculated from NTA agrees with the PDI measured by DLS, indicating that regardless of the orthogonal technique employed, larger sizes and increased heterogeneity were noted when LNPs were manufactured using TM or IJM.

### 3.6. Cryo-TEM Images of LNPs

**Figure 8:**
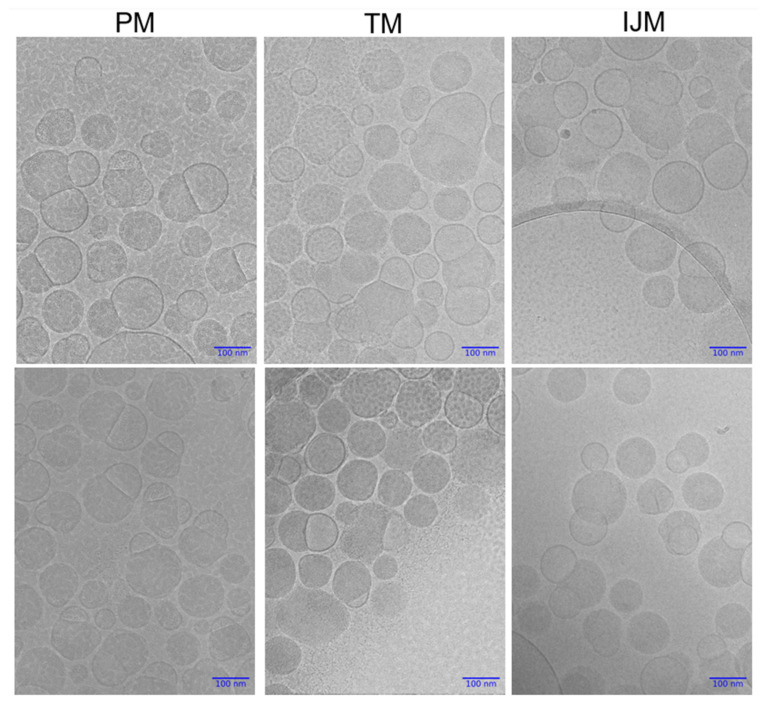
Cryogenic Transmission Electron Microscopy (cryo-TEM) images of Fluc-LNPs manufactured using pipette mixing (PM), T-junction mixing (TM), or impingement jet mixing (IJM). Two images are available for each condition with magnification set at 30,000× with a 100 nm scale bar for all images. Images were captured on JEOL F2-Cryo (200 kV), while image processing was completed using Relion 5.0.0 and Image J.

Cryo-TEM was used to gain further insight into the structure of LNPs prepared by the microfluidic and manual mixing procedures. Figure 8 presents representative cryo-TEM images of LNPs produced via PM, TM, and IJM. All mixing methods produced particles with distinguishable blebs. Across all formulations, particles were observed to contain no blebs, single blebs, or multiple blebs, with no apparent differences in the frequency or distribution of these features between mixing methods.

### 3.7. Exploiting Pipette Mixing as a Cost-Effective High-Throughput Screening Platform

Given that our results demonstrate that pipette mixing can produce small monodisperse LNPs and promote mRNA expression both in vitro and in vivo, we next sought to determine if this approach could effectively discriminate among diverse LNP formulations when used for rapid screening. This would enable its integration into an automated platform for high-throughput formulation development. By systematically assessing key metrics (such as particle size, polydispersity index, and encapsulation efficiency) across multiple pipette-mixed LNP formulations, we aimed to confirm that pipette mixing can provide a practical approach for screening LNP formulations.

**Figure 9:**
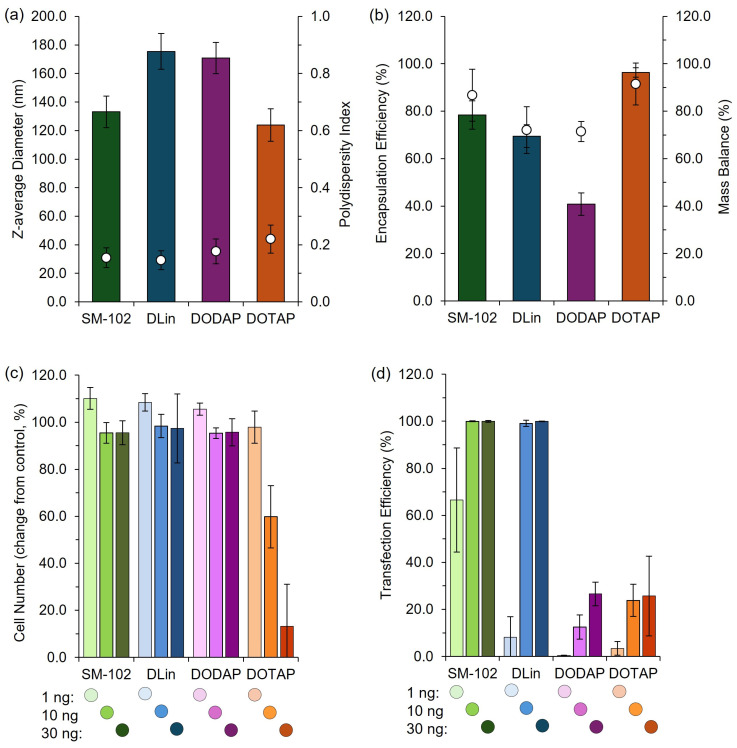
High-throughput LNP formulation and characterization platform. LNPs were formulated using SM-102, DLin-MC3-DMA (DLin), DODAP, or DOTAP as the ionizable or cationic lipid components and prepared using a high-throughput (HT) pipette mixing protocol (see Section 2.15 for details). (**a**) Z-average diameter (bars) and PDI (circles) of LNPs prepared via HT, (**b**) encapsulation and mass balance of LNPs prepared via HT. The LNP efficacy was evaluated by quantifying the change in cell number (**c**) and transfection efficiency (**d**) in a high-throughput cell-based transfection assay. LNPs containing mGreenLantern mRNA were reverse-transfected into a HeLa cell line expressing nuclear-mKate2 at 1 ng, 10 ng, or 30 ng mRNA dose per well on a 384-well plate. Cells were imaged after 42 h incubation using an Opera Phenix high-content screening system and quantified using automated analysis in Harmony. Data are normalized to internal controls. Bars show the mean, and whiskers depict the ± standard deviation.

**Figure 10:**
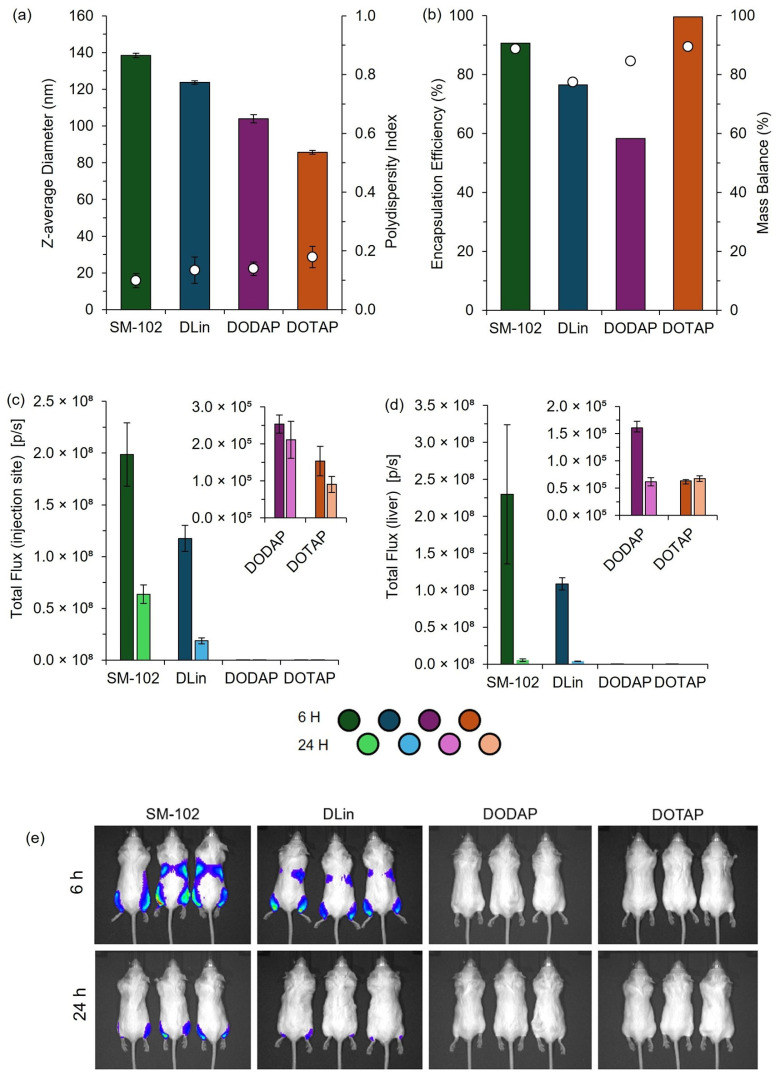
Physicochemical characterization and in vivo performance of mRNA-loaded LNPs prepared by manual pipette mixing. LNPs were formulated using SM-102, DLin-MC3-DMA (DLin), DODAP, or DOTAP as the ionizable or cationic lipid components. (**a**) Particle size represented by the z-average diameter (bars) and polydispersity index (circles), determined by DLS. (**b**) Encapsulation efficiency (bars) and mass balance (circles) were quantified using the RiboGreen assay. In vivo total flux at (**c**) the injection site and (**d**) liver region measured at 6 and 24 h following IM administration in BALB/c mice (dose: 1 µg mRNA per leg). Insets highlight detailed flux data for DODAP and DOTAP formulations. (**e**) Representative bioluminescence images of BALB/c mice at 6 and 24 h post-injection. The color scale indicates the luminescence intensity, ranging from 0.5 × 10^7^ (blue; minimum radiance 2.15 × 10^6^ p/s/cm^2^/sr) to 2.0 × 10^7^ (red; maximum radiance 2.38 × 10^7^ p/s/cm^2^/sr). Data are shown as mean ± SD (size/PDI, *n* = 3 measurements from one batch) or mean ± SEM (in vivo; injection site *n* = 6, liver *n* = 3).

To validate the pipette-mixing approach, we prepared four widely reported LNP formulations, SM102, DLin, DOTAP, and DODAP, each known to exhibit distinct in vivo expression profiles [19]. These formulations were produced in a high-throughput manner using pipette mixing, and their physicochemical characteristics and efficacy were subsequently evaluated in vitro (Figure 9). To finalize this section, we also produced these four formulations using the manual pipette mixing (PM) method to establish key metric differences and evaluate the in vivo efficacy (Figure 10).

These results demonstrate distinct differences in efficacy among the formulations tested, and the expression patterns we obtained were consistent with established profiles in the literature [19], confirming the utility of this rapid, cost-effective pipette-based protocol for laboratory studies and high-throughput screening of LNP formulations. This demonstrates that this high-throughput and manual pipette mixing method can effectively discriminate performance across various LNP candidates in vitro and in pre-clinical mouse models.

## 4. Discussion

Our research demonstrates that low-cost microfluidic mixers can reliably produce LNPs, highlighting their potential as effective tools for affordable small-scale production and high-throughput screening applications. We evaluated three mixing methods: TM, IJM, and PM. While PM may lack precise control over CPPs and is not scalable, it proved effective for small-scale LNP manufacture. Characterization studies showed that CQAs were influenced more by payload (Figure 2) than the mixing method (Figure 3, Figure 4, Figure 5, Figure 6, Figure 7 and Figure 8); for example, poly A yielded smaller LNPs with higher encapsulation efficiencies, whereas mRNA constructs increased particle size. This is likely because poly A is a small, linear molecule compared to mRNA, which can exhibit enhanced secondary structures that may lead to a less tightly packed LNP structure. Cy5-Fluc mRNA also increased the LNP size, possibly due to interactions with the fluorescent tag and lipid components.

We also observed a decrease in encapsulation for LNPs containing Cy5-Fluc (Figure 2), possibly due to the Cy5-tag interfering with the RiboGreen assay. Specifically, Cy5 has an excitation/emission spectrum of approximately 650/670 nm, while Ribogreen operates at approximately 500/525 nm; this spectral overlap suggests that Cy5 has the potential to fluorescently quench the RiboGreen signal, thereby affecting the fluorescence readout. To mitigate this effect, Cy5-Fluc mRNA was used during the assay to build the calibration curves. While not fully quantified in this study, this potential interference should be considered when interpreting encapsulation data involving fluorescently labelled RNA. Fluorescent tags, particularly cyanine dyes, are widely used in LNP and liposomal research and generally do not significantly affect particle characteristics [20]. For example, Ma et al. investigated in vivo mRNA delivery using SM-102-LNPs tagged with Cy5 and DiR, reporting sizes around 100 nm [21]. However, their LNPs were produced at a higher flow rate and purified via dialysis. In contrast, we used spin column purification in our studies, introducing a rapid pH change and high centrifugal forces that may contribute to these differences. Schober et al. also examined the impact of payload size in DLin- and ALC-0315-LNPs, finding no significant effect on size [22]. Therefore, the low-cost mixers tested in our studies may be more sensitive to nucleic acid payload, which may also explain differences in encapsulation efficiency.

It is generally recognized that LNP size is a critical factor in determining biodistribution and transfection efficiency, and previous studies have shown that various mixing methods can significantly influence particle size. To investigate this, we used orthogonal characterization techniques to investigate the physicochemical properties of the LNPs produced by different mixers. DLS measurements showed that PM LNPs had smaller hydrodynamic diameters and lower PDI than TM and IJM mixers (Table 2 and Figure 6). While NTA showed minimal differences in mean particle size across the mixers, PM LNPs displayed a higher particle concentration and narrower size span, indicating a more uniform population (Table 3 and Figure 6). AF4-MALS analysis supported these findings, with PM LNPs exhibiting lower Rg values and narrower elution profiles, indicating a more compact structure (Table 4 and Figure 6). Shape factor analysis (Table 4 and Figure 7) showed all LNPs were generally spherical (Rg/Rh ~0.775), but TM and IJM particles displayed shape factor that increases over time, suggesting some elongation or fusion events. These differences were not visually evident from cryo-TEM images (Figure 8), which showed similar external morphology and bleb features across all samples. This highlights the importance of employing complementary characterization techniques to fully understand LNP behavior, as cryo-TEM alone does not easily show population-level differences.

Our findings are generally consistent with the existing literature regarding the impact of mixer choice. For instance, Misra et al. produced SM-102-LNPs using a confined impingement jet system, reporting sizes around 135 nm with high encapsulation efficiency (>95%), which is smaller than the IJM-generated LNPs in this study [23]. However, these LNPs were manufactured under different conditions and included higher PEG content (2 mol%), which has been shown to reduce LNP size [14,24]. Similarly, Subraveti et al. produced Dlin-MC3-DMA-LNPs using a confined impingement jet mixer, achieving sizes below 100 nm at physiological pH; however, their study also used dialysis purification and had a different lipid composition, which may have contributed to smaller particle sizes [25]. These studies suggest that IJM-generated LNPs can be tuned to smaller sizes by carefully optimizing CPPs, such as the flow rate ratio, lipid composition, and purification method.

Further studies have investigated the impact of size on LNP biodistribution and expression efficiency. Di et al. reported that LNPs ≥ 180 nm exhibited reduced liver expression, while McMillan et al. demonstrated that larger LNPs correlated with significantly decreased in vivo expression [14,26]. Given that TM and PM produced more comparable sizes, they were prioritized for in vivo testing, aligning with previous reports that T-mixed LNPs typically range from 120 to 130 nm [27]. Our results showed no significant difference in vivo expression between the TM and PM LNPs; however, studies comparing T-junction and chaotic mixers suggest that chaotic mixing methods may improve LNP efficiency. For example, Jürgens et al. found that staggered herringbone mixers (SHMs) outperformed T-mixing for mRNA LNPs [27]. This effect could be attributed to chaotic mixers typically producing smaller LNPs, which could increase uptake as size-dependent effects on uptake are generally more pronounced at sizes below 100 nm [28].

There are various reports on the performance of pipette-mixed LNPs. For example, Wang et al. found that pipette-mixed MC3-LNPs had larger sizes (~162 nm) and lower mRNA expression than microfluidic-generated LNPs [29]. In contrast, Strelkova et al. demonstrated that manually mixed LNPs could outperform some microfluidic-generated formulations when process parameters were varied [30]. These results, combined with the results we present, confirm that PM is a useful, low-cost option.

To further validate the use of pipette mixing as part of a larger high-throughput pipette mixing system, both high-throughput and manual pipette mixing were employed to generate clinically relevant cationic and ionizable LNPs for in vitro and in vivo testing. The observed expression profiles aligned with previous reports, with expression increasing from DOTAP > DODAP > DLin > SM-102, consistent with the known efficiency of ionizable lipids (DLin and SM-102) versus cationic lipids (DOTAP and DODAP) in nucleic acid delivery [19]. While PM LNPs exhibited larger sizes, they maintained comparable expression profiles to published data, supporting PM as an effective tool for rapid LNP screening.

## 5. Conclusions

In conclusion, this study demonstrates that low-cost microfluidic mixers can effectively produce LNPs, with pipette mixing (PM) emerging as a viable high-throughput screening tool. While PM, TM, and IJM produced LNPs with similar in vitro expression profiles, PM offers a cost-effective and accessible alternative for small-scale research despite lacking precise control over critical process parameters. Orthogonal analysis confirmed no major physicochemical differences besides size variations, and in vivo testing showed that PM and TM produced comparable expression profiles, reinforcing the potential of using pipette mixing for predicting LNP potency. A high-throughput pipette mixing system was successfully validated, demonstrating that PM can be used for rapid, reproducible LNP screening, supporting its application in early-stage formulation development.

## Figures and Tables

**Table 1:** The zeta potential (mV) of LNPs encapsulating various nucleic acid payloads. Results are representative of mean ± standard deviation (*n* = 3–4).

Mixer	Poly A	Fluc	GrL	Cy5-Fluc
TM	1.6 ± 1.2	0 ± 2.2	2.4 ± 1.9	−0.2 ± 0.6
IJM	0.7 ± 1.3	−0.6 ± 2.2	2.9 ± 1.2	−1.3 ± 2.0
PM	2.3 ± 1.3	1.6 ± 3.1	2.5 ± 2.1	1.3 ± 1.3

**Table 2:** Summary of physicochemical data collected from Zetasizer Ultra (DLS).

Dynamic Light Scattering (DLS)		
Mixers	Z-Average Diameter (nm)	PDI
TM	169.9 ± 5.6	0.199 ± 0.018
IJM	186.4 ± 15.7	0.228 ± 0.035
PM	135.8 ± 5.7	0.112 ± 0.018

**Table 3:** Summary of physicochemical data collected from NanoSight Pro (NTA).

Nanoparticle Tracking Analysis (NTA)							
Mixers	Mean (nm)	Mode (nm)	D10 (nm)	D50 (nm)	D90 (nm)	Span	Concentration (Particles/mL)
TM	113.2 ± 5.3	101.5 ± 6.3	76.2 ± 3.9	107.3 ± 5.5	156.8 ± 8.8	0.75	7.06 × 10^11^
IJM	115.1 ± 4.6	99.6 ± 9.0	77.8 ± 2.8	109.6 ± 5.0	158.3 ± 5.7	0.73	6.87 × 10^11^
PM	113.4 ± 8.3	101.8 ± 10.0	77.5 ± 6.5	108.4 ± 78	154.0 ± 10.9	0.71	1.35 × 10^12^

**Table 4:** Summary of physicochemical data collected from AF4-MALS.

AF4-MALS							
Mixers	Et-(min)	Rec (%)	Rg Mode (nm)	Rg-D10 (nm)	Rg-D50 (nm)	Rg-D90 (nm)	Rg-Span
TM	37.5 ± 2.4	79.1 ± 8.8	60.3 ± 4.9	44.1	59.1	72.2	0.48
IJM	39.2 ± 1.7	90.0 ± 22.0	63.1 ± 2.3	46.1	62.4	77.1	0.50
PM	32.8 ± 0.1	96.4 ± 7.8	47.4 ± 0.4	39.3	46.5	56.6	0.37

## Data Availability

The data generated will be made available from the corresponding author on reasonable request.

## Supplementary Materials

The following supporting information can be downloaded at: https://www.mdpi.com/article/10.3390/pharmaceutics17050566/s1, Figure S1 Gating Strategy for unstained live cell control; Figure S2: Gating Strategy for stained live cell control; Figure S3: Gating Strategy for stained dead cell control.

## Abbreviations

The following abbreviations are used in this manuscript:

LNP	Lipid nanoparticle
PM	Pipette mixing
TM	T-junction mixer
IJM	Confined impingement jet mixer
DoE	Design of Experiments
CQA	Critical quality attribute
CPP	Critical process parameter
SHM	Staggered herringbone mixer
TFR	Total flow rate
FRR	Flow rate ratio
Fluc	Firefly Luciferase mRNA
GrL	Green Lantern mRNA
DLS	Dynamic Light Scattering
NTA	Nanoparticle Tracking Analysis
AF4	Asymmetric flow field-flow fractionation
MALS	Multiangle light scattering
Rg	Radius of gyration
Rh	Hydrodynamic radius
