# Supplementary material for: Low-Cost Microfluidic Mixers: Are They up to the Task?
Source: Pharmaceutics. 2025 Apr 25;17(5):566. doi: 10.3390/pharmaceutics17050566 (PMC12115082; doi:10.3390/pharmaceutics17050566)

Supplementary Information

Low-Cost Microfluidic Mixers: Are They Up to the Task?

Jade C. Forrester, Callum G. Davidson, May Blair, Lynn Donlon, Daragh M. McLoughlin, Chukwuebuka R. Obiora, Heather Stockdale, Ben Thomas, Martina Nutman, Sarah Brockbank, Zahra Rattray and Yvonne Perrie.

Flow cytometry analysis was gated based on three control groups: unstained live cells (Figure S1), stained live cells (Figure S2), and stained dead cells (Figure S3). Once gating was appropriate for all three controls, sample analysis was undertaken.

Figure S1 Gating Strategy for unstained live cell control. Gating strategies were set up using an unstained live cell control (shown below) before being used for positive and negative controls as well as sample runs. (A) All Cells gate (red) was set to contain the live and healthy cell population and exclude any dead cells or possible cell debris as indicating by very high or low FSC-A and SSC-A (B) single cells gate (blue) were selected to exclude cells populations that were possibly stuck together as indicated by an increase in one of either side scatter area or height (C) BL2 voltages were adjusted until complete signal seen on the left hand side of the panel within live cells gate (yellow) indicating cell viability as propidium iodide stains dead cells while (D) RL1 voltages were adjusted until Cy5+ signal gate (green) had minimal signal remaining on the right had side of panel, finally (E) represents Cy5+ signal as a function of forward scattering.

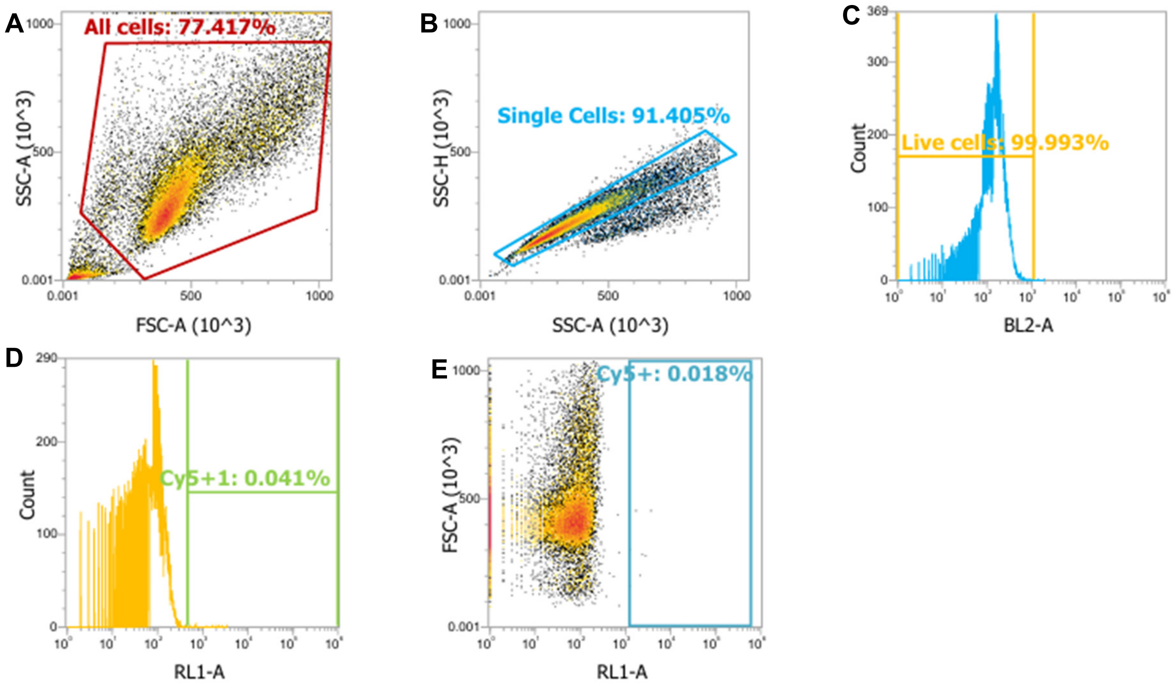

Figure S2. Gating strategy for stained live cell control. Gating strategies were established using a stained live cell control (shown below) prior to sample analysis. All gates (A–E) were applied as described in Figure S1, using these control samples to confirm that the cell population remained viable following sample preparation. This ensured that results were representative of a live, healthy cell population.

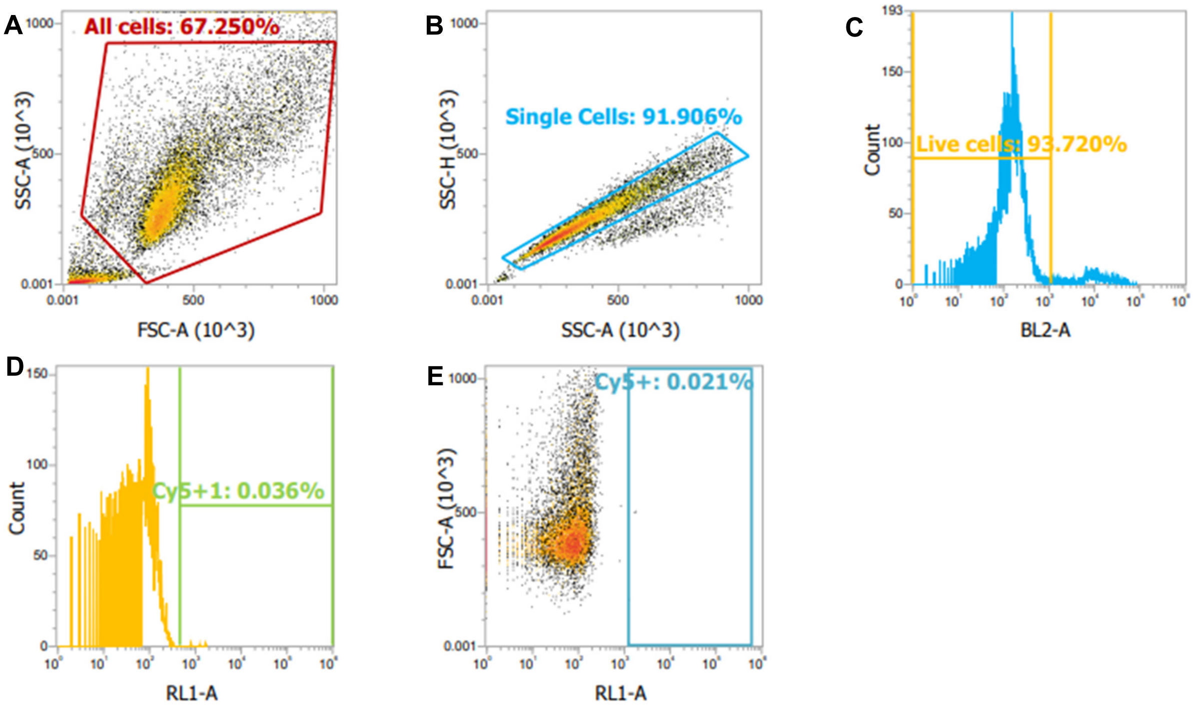

Figure S3 Gating Strategy for stained dead cell control. Gating strategies were set up using a stained dead cell control (shown below) before being used for sample runs. All gates (A-B, D-E) were set up as described for Figure S1 using these dead cell samples to help ensure that the gating was correct and ensure viability. For the live cells gate (yellow) (C), all signals should be present on the right-hand side of the panel due to the propidium iodide signal being positive and staining dead cells.

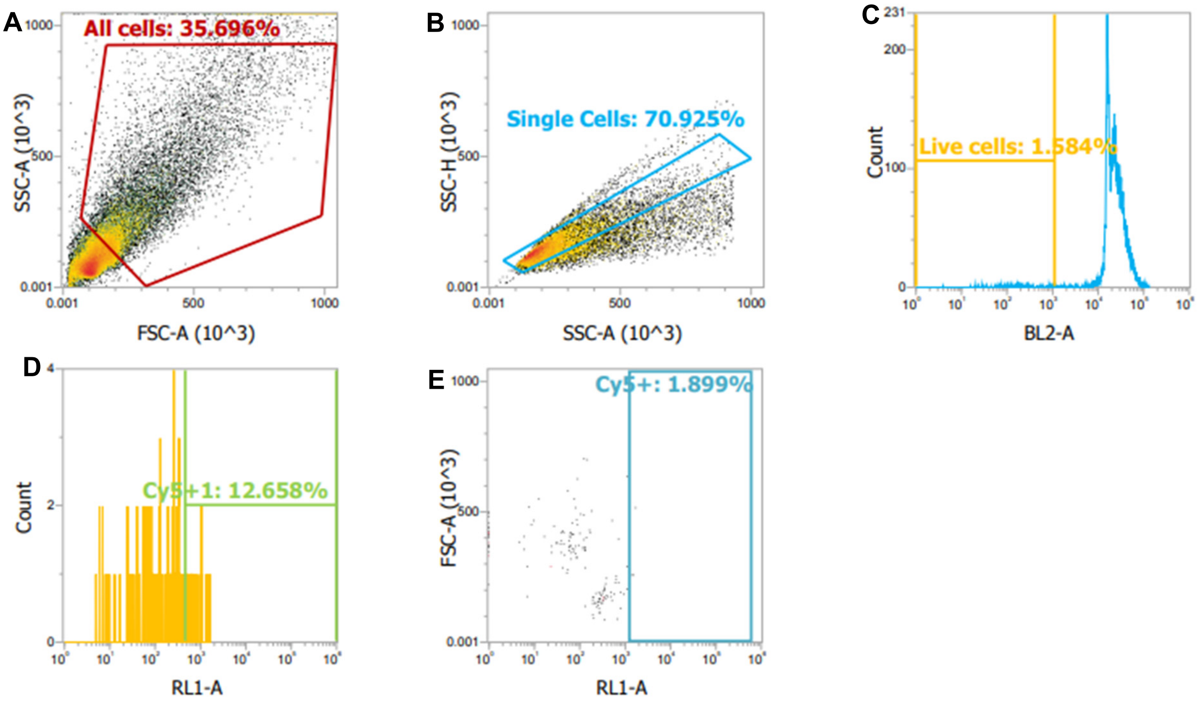

Supplement: Supplementary file 1 [file pharmaceutics-17-00566-s001.zip › pharmaceutics-3572232-supplementary.pdf]
